# ESPRESS.0: Eustachian Tube-Inspired Tactile Sensor Exploiting Pneumatics for Range Extension and SenSitivity Tuning

**DOI:** 10.3390/s23020567

**Published:** 2023-01-04

**Authors:** George P. Jenkinson, Andrew T. Conn, Antonia Tzemanaki

**Affiliations:** Department of Mechanical Engineering, University of Bristol, Bristol BS8 1TR, UK

**Keywords:** pneumatic, soft robotics, tactile sensors, variable stiffness, medical palpation

## Abstract

Optimising the sensitivity of a tactile sensor to a specific range of stimuli magnitude usually compromises the sensor’s widespread usage. This paper presents a novel soft tactile sensor capable of dynamically tuning its stiffness for enhanced sensitivity across a range of applied forces, taking inspiration from the Eustachian tube in the mammalian ear. The sensor exploits an adjustable pneumatic back pressure to control the effective stiffness of its 20 mm diameter elastomer interface. An internally translocated fluid is coupled to the membrane and optically tracked to measure physical interactions at the interface. The sensor can be actuated by pneumatic pressure to dynamically adjust its stiffness. It is demonstrated to detect forces as small as 0.012 N, and to be sensitive to a difference of 0.006 N in the force range of 35 to 40 N. The sensor is demonstrated to be capable of detecting tactile cues on the surface of objects in the sub-millimetre scale. It is able to adapt its compliance to increase its ability for distinguishing between stimuli with similar stiffnesses (0.181 N/mm difference) over a large range (0.1 to 1.1 N/mm) from only a 0.6 mm deep palpation. The sensor is intended to interact comfortably with skin, and the feasibility of its use in palpating tissue in search of hard inclusions is demonstrated by locating and estimating the size of a synthetic hard node embedded 20 mm deep in a soft silicone sample. The results suggest that the sensor is a good candidate for tactile tasks involving unpredictable or unknown stimuli.

## 1. Introduction

Tactile examination of an object requires a physical interaction between a tactile sensor and the object that is its stimulus. As such, the interpretation of the signals from a tactile sensor must consider the sensor and the stimulus to be a unified physical system [1]. This is in contrast to other sensory systems, such as audio and visual sensors, where the sensor does not affect the stimulus. Humans have mechanoreceptors located within the muscles used to actuate joints, such that the actuation state of the muscles, as well as any other sensory feedback, contributes to the haptic perception of the environment [2]. By mapping an applied force to an observed deformation, a distinction can be made between different materials of various stiffness levels, which physicians use to identify and characterise palpable lumps within human tissue [3,4].

Palpating to locate and characterise tumours or other stiff nodes during surgery or a medical examination allows medical professionals to begin diagnosis, plan for surgical procedures, and track the progress of known malignant tumours [5,6]. Precision, repeatability and accuracy are of paramount importance in these tactile medical examinations to ensure favourable patient outcomes. A range of automatic and semi-automatic devices have been proposed to aid with optimising this task, particularly for difficult to detect and hard to reach situations such as during minimally invasive surgery [5,7].

Semi-automatic devices with a human-in-the loop controller can take advantage of the user’s ability to palpate effectively and may be used to guide a set of wearable robotic sensors, e.g., mounted to a glove, whose output can be recorded and analysed to provide insight into the properties of the tissue [8]. Similarly, a human controlled remote system may allow a human operator to palpate tissue that would otherwise be inaccessible [9]. However, tactile instruments used to detect stiff nodes in soft biological tissue have been shown to consistently achieve higher detection rates whilst using lower forces when they are operated by a robot controller, compared to being operated by a human manipulating the same instrument [10]. This suggests that an automatic device would yield more reliable results whilst applying lower forces, and so is less likely to cause damage and be more comfortable for an examinee compared to a human controlled system. However, the failure of fully automated devices to match the adaptability of a human operator has prevented the widespread uptake of automated devices for palpation purposes.

Automated palpation by a robotic device is challenging, and many feed-forward devices with static mechanical properties have been demonstrated to yield inadequate information to be medically useful [11]. Closed-loop feedback control mechanisms that employ active touch to optimise exploratory palpation yield higher quality tactile information [12], yet are usually limited by the mechanical properties of their constituent materials, such as the elastic modulus. Emerging closed-loop control schemes for human–robot interaction which actively control stiffness such as variable impedance control can suffer from stability issues [13]. It is, therefore, hard to achieve high quality results for a palpatory task involving unpredictable tactile stimuli that may exhibit a large range of stiffnesses when controlling only the force, position, and velocity of the sensor. Soft robotic sensors that can actively control their inner pneumatic pressure, and therefore their effective stiffness, shape, and friction coefficient have demonstrated utility in a number of tactile tasks including estimating bulk tissue elastic modulus [14,15], detecting the presence of an embedded stiff node in a soft material [16], and detecting and reacting to slip [17,18].

Controlling the rotational stiffness and inertia of a finger-like robotic probe has been shown to enhance its sensory capabilities when estimating node depth and hardness during palpation examinations of soft tissue [2,19]. This is because a ‘stiffening’ actuator may control the sensitive range available to a sensor it is linked to, which maximises its sensitivity as is appropriate to the stimulus. Pneumatic pressure modulation of a soft sensor membrane has also been used as a mechanism to increase the sensor’s stiffness to help improve the tactile sensing performance across a variety of tactile applications [20,21,22], with specific benefits towards enhancing edge detection and reducing `cross-talk’ between sensory units [17,23].

Automated control for palpatory tasks often takes inspiration from the human hand and arm, leading to the development of robotic devices that modulate their rotational stiffness in such a manner. Conversely, the mammalian eardrum and middle ear make a biological sensory system that exhibits control over its effective stiffness. A difference in pressure between the enclosed air in the middle ear and the outside air results in a force over the eardrum, causing it to stiffen and therefore increases its resonant frequency. This alters the impedance match with the outside air which carries the stimulus, impairing the functionality of the sensory system [24,25]. To mitigate this, the pressure of the middle ear is periodically matched with the ambient pressure of the outer environment via opening and closing the Eustachian Tube, maximising the sensitivity of the sensing organ to its stimulus [25,26].

Soter et al. demonstrated a mechanism where tactile information can be translocated using an incompressible liquid and measured by an optical sensor at a location away from the tactile interface [27]. Coupling a compliant tactile interface to a sensor using an incompressible liquid allows a near lossless transfer of energy by transmitting pressure from one end of the liquid to the other. Following the same principle, a controlled pressure may be applied at the end of the liquid away from the compliant tactile interface to adjust its compliance in order to have the appropriate sensitivity for its stimulus. The contribution of enclosed pressurised air to the compliance of this mechanism was explored theoretically in our previous work [28], where it was theorised that adaptive control over a soft membrane’s stiffness using a pneumatic compressor could be used to optimise its sensitivity ad hoc as appropriate to a particular task. The results showed that having a stiffer, higher pressure air reservoir sacrifices sensitivity at small forces for a higher sensitivity at large forces, with an increased sensitive range. The advantage of elastomeric tactile sensors with pneumatically tunable stiffness for adaptive sensitivity has recently been realised in a pressure based [29] configuration, and the coupling of silicone to an incompressible conductive liquid of variable pressure has been realised to construct a conductivity based [30] soft sensor.

Robotic palpation devices and platforms in the literature do not include tumour-mimicking inclusions that exceed a depth of 15 mm [3,5,7,19,31,32,33,34,35,36,37]. This illustrates a particular gap when it comes to carrying out Clinical Breast Examinations, where the depth of a tumour may exceed 15 mm [38]. Further, high node heterogeneity may be an important diagnostic feature for breast cancers [39,40,41,42], meaning that intra-node stiffness is likely to vary and, hence, detecting tactile cues at sub-tumour size scales becomes a useful property that has not been previously developed.

This paper presents the Eustachian tube-inspired Sensor exploiting Pneumatics for Range Extension and SenSitivity tuning (ESPRESS.0), a tactile sensor that applies the principle of stiffness adjustment in a manner analogous to the mammalian eardrum. The translocation of fluid within the ESPRESS.0 allows it to transduce a tactile input into an optically measurable output, with a similar principle to Soter et al.’s SkinFlow sensor [27], and a pneumatic back pressure enables dynamically tunable stiffness and improved sensitivity. The ESPRESS.0 has been designed incorporating a soft membrane with the specific aim of safe interaction with skin and making it a candidate for palpating tissue to search for stiff nodes.

The structure of the paper is as follows: Section 2 sets out the the practical design of the sensor Section 3 qualitatively describes the operational modalities of the sensor, details the experiments carried out to characterise the sensor, and includes a model for the sensor. Section 4 outlines the experimental evaluation for the actuator-sensor and presents the results. Section 5 includes a discussion and considerations for further work. Section 6 summarises the conclusions drawn from the paper.

## 2. Methods

### 2.1. Design and Fabrication

#### 2.1.1. ESPRESS.0

**Figure 1:**
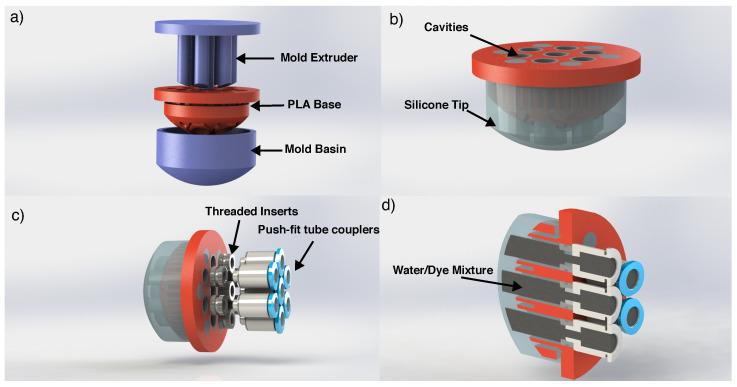
CAD views of the ESPRESS.0 fabrication process. (**a**) An uncured silicone mixture is added to the mould basin, the base and extruder are then clamped onto the basin. The mould parts (blue) may be used to fabricate another ESPRESS.0 tip. (**b**) The silicone cures in the negative space, leaving cavities in the tip. (**c**) The tip is prepared for watertight coupling to 2 mm OD tubing. (**d**) Cross-section showing the cavities filled with the water/dye mixture before silicone tubing is attached.

The ESPRESS.0 has a silicone tip whose geometry is comprised of a spherical cap with a 24 mm outer diameter, capped such that its geometric base is a 20 mm diameter circle, attached to a rigid plastic (PLA) base (Figure 1).

**Figure 2:**
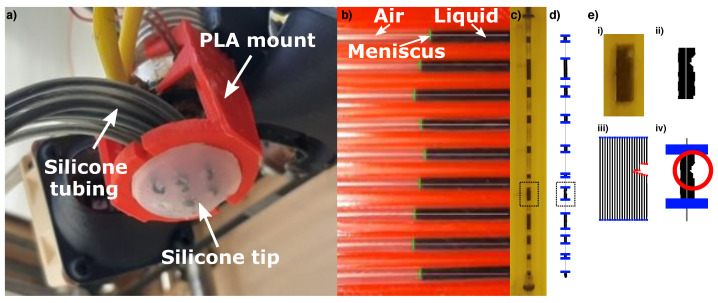
(**a**) The ESPRESS.0 mounted to a Cartesian arm, with the tubes channelled to pass in front of a camera. (**b**) The camera monitors the position of the liquid the channels against a red housing. (**c**,**d**) A single channel against a yellow housing has multiple menisci to increase the tracking range and smooth any noise. The image is processed to produce the menisci positions in blue. (**e**) Marked section of c and d showing the process (i) from camera frame (ii) converted to monochrome (iii) splitting to 20 vertical strips and finding edges (iv) to filtering out noise and artefacts.

To fabricate an ESPRESS.0 tip, a 3D printed mould basin is filled with silicone mixture (Dragon Skin™ 10 medium, Smooth-On), before the 3D printed PLA base and mould extruder are clamped onto the basin, leaving the silicone to cure in the negative space (Figure 1). The extruder is removed and threaded inserts are added so that a pneumatic fitting can couple tubes to each of the cavities left in the silicone to serve as the mechanoreceptive regions. The cavities and tubing are filled with water with black food colouring 1:1 ratio by volume. The tubes transmit the movement of the liquid (due to compression at the silicone interface) to where it can be observed by a camera (Figure 2) (after [27]). In this way, the camera is remote with respect to the area of physical contact, and so is protected from damage while the dome remains portable.

A thin silicone membrane separates the liquid filled cavity and the outer layer of the tip. This membrane acts as the device’s tactile interface and its mechanical properties affect the sensor’s characteristics. The thickness of the membrane is fixed during fabrication, but the mechanical properties may also be adapted ad hoc by changing the internal pressure of the ESPRESS.0 sensor. The nominal pressure of the ESPRESS.0 (the internal pressure when the silicone tip is not in contact with an object) can be set by controlling the volume of the air in the channels. Here, an Arduino DUE is used to control a syringe driver to set the nominal pressure. The syringe driver consists of a stepper motor (180-5290, RS Components, Corby, UK) which drives the plunger of a syringe along 2 linear slides until the desired nominal pressure state is reached. The membrane and the nominal pressure combine to define the sensor’s characteristic response to an applied force (explored in Section 3.4).

Examining tissue for nodes becomes useful from the perspective of detecting signs of possible early-onset cancerous growths when nodes with a size in the region of 10–20 mm can be detected [43]. For this purpose, the device has been fabricated to include 7 cavities of 4 mm diameter, each with its centre 5 mm from its nearest neighbours’, arranged in a hexagon around a central node, each connected to a cylindrical silicone tube of 1 mm inner diameter (Figure 1). The focus of this paper is to characterise the sensory response of a single sensory channel, and so all results demonstrated herein correspond to the central cavity of the sensor. However, the 7-cavity layout demonstrates that a sensor tip with multiple channels is simple to fabricate, and having a single optical sensor that capture multiple channels means that the system can easily be scaled to have a denser collection of smaller sensitive regions, or expanded to be comprised of more sensitive regions over a larger surface without loss of sensitivity or operational speed.

#### 2.1.2. Untethered Version

**Figure 3:**
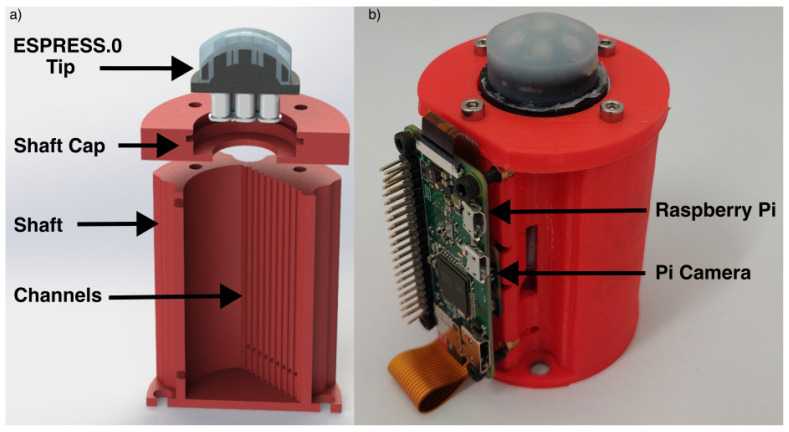
The untethered ESPRESS.0’s channels are concealed in an LED-lit shaft, and the data is streamed by a Raspberry Pi via WIFI. (**a**) Exploded CAD cross-section showing concealed channels. (**b**) Untethered ESPRESS.0.

To increase ease of adaptation and integration with other systems, an untethered version of ESPRESS.0 with reduced functionality is demonstrated herein. The untethered ESPRESS.0 does not have the ability to adjust its internal pressure, greatly reducing its mass and increasing portability. An ESPRESS.0 tip, Raspberry Pi and camera were mounted to a PLA shaft (Figure 3), the channels were each partly filled then sealed. A Raspberry Pi and camera were fixed opposing the liquid channels, and the Raspberry Pi was calibrated to stream its video over WIFI to a connected PC for analysis.

### 2.2. Image Processing

The objective of the image processing is to track the position of the meniscus as it moves. The silicone tubing is channelled to pass through a light coloured PLA housing, where the black liquid in the tubes contrasts strongly with the PLA background. Each RGB image frame captured by the camera was converted to a monochrome image using a threshold value with the OpenCV library [44] for analysis. The PLA housing fixes the position of the camera and channels with respect to each other, so that known vertical sections of the image relating to each channel can be analysed separately. The menisci may move significantly enough to be out of the field of view of the camera. Because of this, small air bubbles were deliberately introduced throughout the liquid by rapidly alternating the flow of the liquid in the tube to create a turbulent flow at its meniscus, allowing the ingress of air into the liquid. This created multiple stable menisci for the camera to track (Figure 2c). Due to hydraulic pressure transmission, pressure is equalised across the controlled pressure compartment and all of the air bubbles. The volume of the air bubbles is negligible compared to the pressurised air reservoir, such that the compression of the bubbles due to a change in pressure caused by a force applied at the membrane causes no measurable volume change in the bubbles.

For each channel, an edge finding filter that looks for contrasting neighbouring pixels is passed over the areas of each frame corresponding to that channel. The channels are each typically 20 pixels wide and 2000 pixels high in the captured video, such that the filter can be passed over the area vertically 20 times. If more than half of these passes detect a line at the same location (within a tolerance of 1 pixel up/down), then that location is associated with a meniscus position that can be tracked over time (Figure 2d,e). Optical noise (reflections, stains) may be removed by grouping the data from consecutive frames and identifying artificial edges as those that do not move when others do, and those that do not exist consistently over time.

In this way, the processing gives a series of coordinates which describe the 1-dimensional movement of the liquid along each tube channel. A pixel in the centre of the image in this set-up corresponds to an area of 32.8±16.4 μm × 32.8 ± 16.4 μm.

Using a camera (Samsung, 2160 × 3840, 30FPS, Folded Type) and image processing means that multiple channels can be monitored at once, reducing the complexity of the sensor and making scaling simple [27].

## 3. Characterisation and Modelling

A controlled environment where the pressure, angle of contact, and force of contact are measured is necessary to characterise the properties of the sensor system. The nominal pressure of the ESPRESS.0 is defined as the internal pressure when the silicone tip is not in contact with an object. As the system is sealed and the temperature does not significantly change during operation, the syringe position can be associated with a nominal pressure state, providing a label for the mechanical response of the membrane from that state.

**Figure 4:**
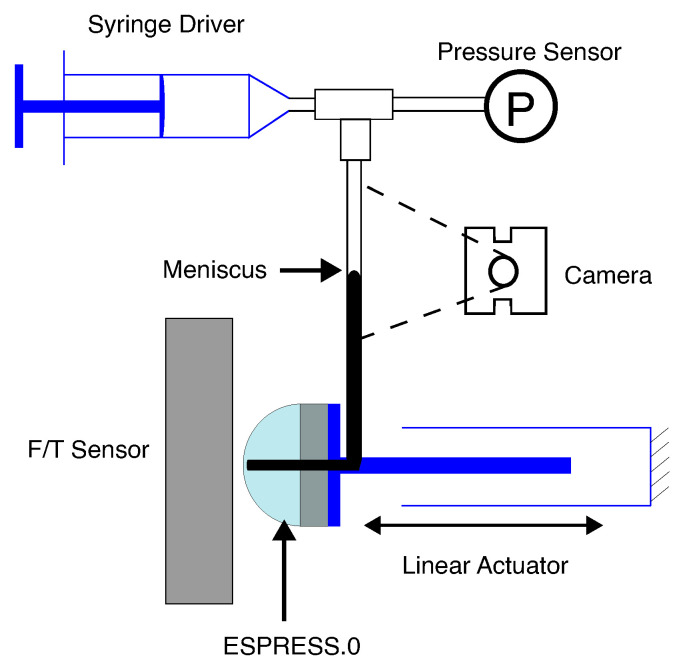
The setup of the ESPRESS.0 prototype with a single channel used to characterise the sensory response. The tubing is all linked, with a 3-way junction adding a pressure sensor.

For characterisation, a pressure sensor (ABPDANV030PGAA5, Honeywell, Charlotte, NC, USA) was added to the syringe driver to give a tight closed-loop control over the nominal pressure of the system, and a linear driver was added to the Arduino to control the axial position of the ESPRESS.0 tip. The linear driver consists of a stepper motor (QMot QSH6018, Trinamic, Hamburg, Germany) which drives the ESPRESS.0 sensor along 2 linear slides to a set distance, with feedback from an optical encoder (TMCS-40, Trinamic, Hamburg, Germany). Running these systems together gives precise control over position and the nominal pressure of the system (Figure 4).

Separating the actuation (pressure and position) and sensing (optical) modalities in this way allows each to operate independently and so simplifies the control and signal processing.

### 3.1. Mechanical Impression

**Figure 5:**
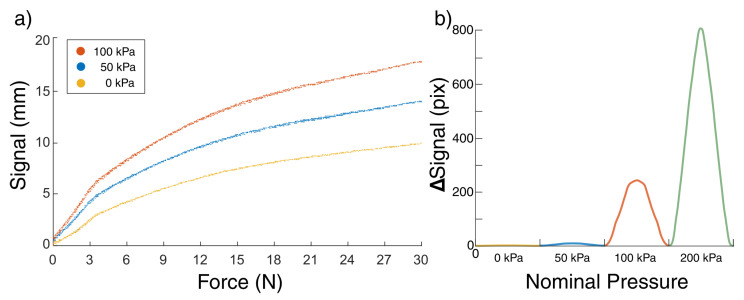
Changing behaviour when applying an internal pressure to a 2.5 mm sensitive membrane. (**a**) The signal (observable movement of the meniscus) is significantly increased when the back-pressure is doubled. The graph shows all data collected over 10 palpations of the F/T sensor at 3 nominal pressures. A function was fitted to each of these curves so that the force could be calculated from the $P_{0}$ and signal. Under 3 N, the three states of nominal pressures 0, 50, and 100 kPa have approximately constant stiffnesses of 2.02, 1.18, and 0.56 N/mm, respectively. (**b**) 1 s oscillations between 35 and 40 N. At high forces, a back pressure can be applied ad hoc to recover sensitivity. With no stimulus present, a 200 kPa internal nominal pressure is likely to break the silicone membrane.

For calibration of the ESPRESS.0 sensor, a Force/Torque (F/T) sensor (Axia80-M8, ATI, Apex, NC, USA) was held by a 3D printed ABS jig perpendicular to the axial direction of the linear motor controlling the ESPRESS.0 tip’s position. This allowed the position of the liquid meniscus to be mapped to a measured force, whilst simultaneously characterising the stiffness of the ESPRESS.0 at various back-pressures (Figure 4). This process was repeated for nominal pressures of 0 kPa, 50 kPa, and 100 kPa above atmospheric, so that each pressure could be associated with its own characteristic curve (Figure 5).

The pressure sensor used for the experimental characterisation is a viable means to sense interactions at the elastomeric membrane. The pressure change induced by an applied force has a large dependence on the volume of the air in the system, a force applied to a large volume of air results in pressure changes that may be less than the noise in a typical pressure sensor [28]. One instantiation could be to completely drain air from the system and have a completely hydraulic sensor. This results in a sensor with a very high sensitivity to both magnitude and temporal resolution of an applied force. However, such a system would scale poorly, needing a pressure sensor for each sensitive channel and so is both expensive and susceptible to failure compared to using an optical sensor.

The smallest stimulus that gives a stable signal response in the range of 0N corresponds to a change of 0.012 N for the range of back-pressures characterised in this study, where the movement of the meniscus by a single pixel correlates to 0.012 N, and the noise in the signal from a static stimulus is smaller than a pixel.

### 3.2. Sensitivity Recovery

An ESPRESS.0 tip not connected to a pressure supply has a limited range, and a static range of forces that it is most sensitive to. Once an ESPRESS.0 tip that is connected to an pressure supply has been compressed such that it is no longer working in its optimal range, its back-pressure can be increased ad hoc to recover sensitivity.

The signal from the ESPRESS.0 in the nominal pressure state of 100 kPa diminishes for contact forces upwards of 30 N. By increasing the internal pressure once ESPRESS.0 is in contact with a force in this range, sensitivity is recovered and the ESPRESS.0 can continue collecting data in a higher force range (Figure 5b). If the nominal pressure of 200 kPa had been initially set before contact, the pressure over the membrane would have been sufficient for it to rupture, so this is an exclusively an ad hoc mechanism. The pressure recovery is effective enough that the sensitivity of the 200 kPa ESPRESS.0 in this range exceeds the sensitivity around the 0 N range, at 0.006 N/px.

### 3.3. Noise Characterisation

Over significantly long timescales the water-based dye may evaporate slightly, causing a drift in the sensor. As the dye is easily replenishable, and this effect is not present on timescales appropriate for most tactile tasks, measured the drift and hysteresis were measured over 90 min. The applied pressure was cycled between 100 kPa and back to its original state such that the pressure changed each minute over this period. These pressure states were associated with meniscus positions at a distance of 49 mm from each other. The meniscus returned to the original position with an accuracy of 0.25 mm in 42 out of the 45 cycles (σ=0.0933), and the final reading was equal to the initial reading within the camera’s resolution relating to 32.8±16.4 μm, demonstrating that the system did not exhibit any significant drift over an order of time comparable to the experiments.

### 3.4. Mechanical Modelling of Membrane

The back-pressure applied to the interior of the membrane results in a force resolved over the membrane. A hoop stress is generated in the membrane as it expands spherically. The relevant parameters for the membrane are the material and the thickness, so multiple ESPRESS.0 sensors were made with a range of membrane thicknesses.

The elastomer membrane has principal stretches $\lambda_{1}$, $\lambda_{2}$ and $\lambda_{3}$ and can be assumed to be incompressible ($\lambda_{1}\lambda_{2}\lambda_{3}=1$) (after [45]). The spherical membrane exhibits axi-symmetric deformation under internal pressure and when interacting with co-axial stimuli. Modelling off-axis stimulus loading is outside the scope of the present study. Therefore, the in-plane stretches, $\lambda_{1}$ and $\lambda_{2}$, are equal, and can be considered as the biaxial stretch λ, which leads to following relationship with the transverse stretch $\lambda_{3}$

$$\begin{array}{cc} \; & \lambda_{1}=\lambda_{2}=\lambda \Rightarrow \lambda^{2}=\frac{1}{\lambda_{3}} \end{array}$$

The biaxial stretch of the membrane is the ratio between the current and initial surface area

$$\lambda^{2}=\frac{2\pi Rh}{\pi r_{0}^{2}}$$

where *R* is the radius of curvature of the membrane, $r_{0}$ the nominal radius of the membrane, and *h* is the pole height. For a spherical cap

(1) $$R=\frac{r_{0}^{2}+h^{2}}{2h}\Rightarrow \lambda^{2}=1+\frac{h^{2}}{r_{0}^{2}}$$

which allows the membrane thickness *t* to be expressed as a function of biaxial stretch λ and initial thickness $t_{0}$

(2) $$t=t_{0}\lambda_{3}=\frac{t_{0}}{\lambda^{2}}$$

The thin membrane deforms spherically, so the hoop stresses in the membrane can be equated to the internal pressure using the thin-walled assumption and Equation (2)

(3) $$\sigma_{1}=\sigma_{2}=\sigma =\frac{PR}{2t}=\frac{PR\lambda^{2}}{2t_{0}}$$

where σ is the biaxial in-plane stress and *P* is the pressure difference across it.

**Figure 6:**
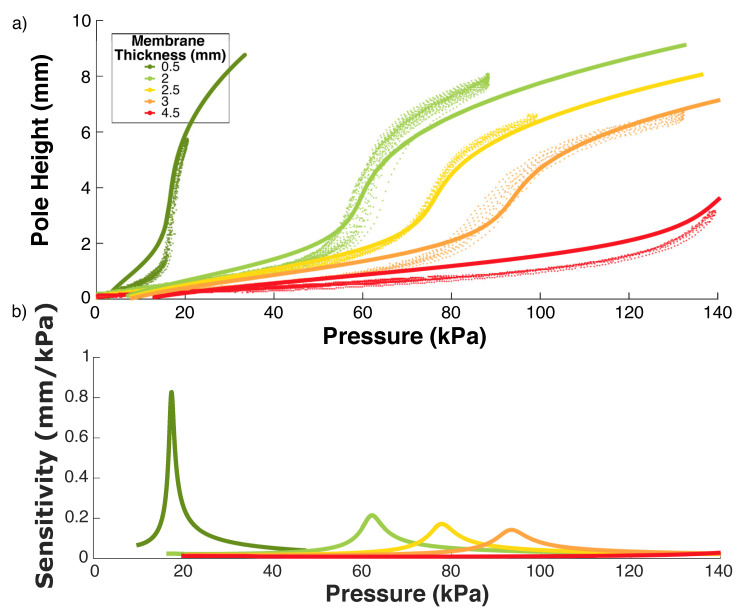
(**a**) Experimental data (markers) and modelled curves (solid lines) with Ogden parameters $\mu_{1}=911$, $\alpha_{1}=5.88$, $\mu_{2}$ = 37,500, $\alpha_{1}=1.45$ [46]. (**b**) Examination of the sensitivity of the membranes for each thickness tested. The membrane has the largest volume change in reaction to an applied force during the steepest stage of the expansion.

The Ogden hyper-elastic model [45] describes the stress–strain relationship for isotropic, incompressible and strain rate independent materials. Using the Ogden model for σ in Equation (3) gives

(4) $$\frac{PR}{2t_{0}}=\sum_{n=1}^{N}\mu_{n}\left(\lambda^{\alpha_{n}-2}-\lambda^{-2\alpha_{n}-2}\right)$$

where μ and α are fitted parameters. Equation (4) can be combined with Equation (1) to predict *h* for an applied pressure (Figure 6).

The behaviour of the material can be sufficiently captured using the 2nd degree Ogden model, rather than the more complex 3rd degree [46,47], reducing the number of parameters to be found to 4. The following values for Dragonskin 10 from [46] were used: $\mu_{1}=911$, $\alpha_{1}=5.88$, $\mu_{2}$ = 37,500, $\alpha_{1}=1.45$.

**Figure 7:**
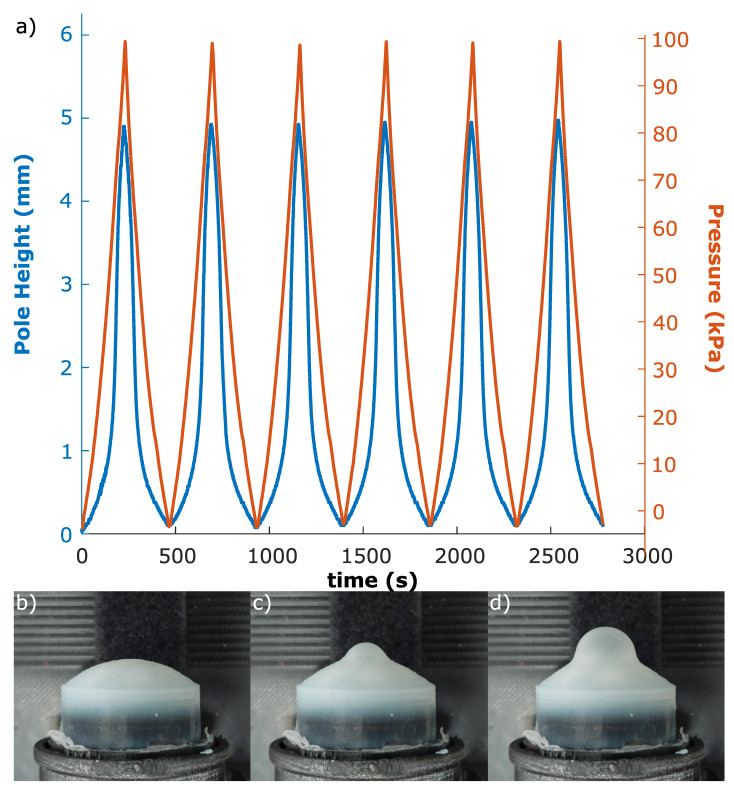
(**a**) Synchronised optical tracking and pressure data for six repeated cycles between high and low pressure on a 2.5 mm membrane. The similarity of the cycles indicates that the response is repeatable. (**b**) Low, (**c**) medium, and (**d**) high pressure state stills of a 2.5 mm membrane.

Experimental data were gathered using a syringe driver (Section 3) cycling between atmospheric and a high pressure while the volumetric change of the elastomer was observed by a camera (Figure 7). The high pressure was determined either at 140 KPa, or at a pressure significantly lower than that which would cause the elastomer to rupture.

The steepest section of the function plotted in Figure 6 relates to the most sensitive pressure range for that membrane thickness in air, where a small compressive force results in a large volume change. This means that at around 90 kPa, an ESPRESS.0 with membrane thickness 2.5 mm will be maximally sensitive to an object’s surface.

## 4. Experimental Evaluation

To demonstrate the aptitude of ESPRESS.0 to sensory tasks, here we evaluate ESPRESS.0’s performance in 3 distinct tasks: Shape reconstruction, where the sensory data is used to reconstruct hard and soft objects; Stiffness classification, where the feedback is used to classify the state of a platform that exhibits a range of controlled stiffness values; and constructing a 3D tactile map of synthetic tissue, where the untethered ESPRESS.0 palpates synthetic tissue to locate and estimate the position and shape of an embedded hard object.

### 4.1. Shape Reconstruction

The initial stage of an automated exploratory palpation task is to find where the stimulus lies in 3D space. Here, we demonstrate that the ESPRESS.0 has the requisite sensitivity to perform an accurate high-resolution shape reconstruction, and employ a simple strategy to do so.

The ESPRESS.0 was mounted to a 3DOF xyz cartesian gimbal, which carried out a series of compressions in the z direction, such that the sensor did not move laterally while in contact with a stimulus. Thus, the xy resolution of a single palpation pass is limited by the diameter of the tip’s channels. If this were of importance in specific applications, the diameter could be reduced in order to include more of them in the tip at the fabrication stage. Here, we use multiple passes with a known distance between them to achieve a super-resolution of 0.5 mm, defined by the distance that the Cartesian robot moves in the xy plane between each pass.

**Figure 8:**
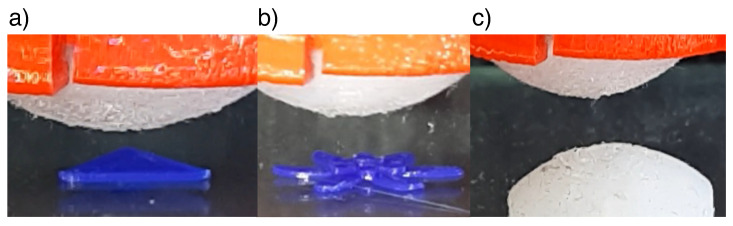
From above, the ESPRESS.0 sensor palpates: (**a**) a hard triangle of 0.3 mm height and 5mm edges; (**b**) a multi-level petal model with heights of 0.5 and 1 mm, and maximum width of 10 mm; (**c**) a soft dome of diameter 12 mm from above. Each palpation consists of multiple passes in a grid separated by 0.5 mm.

**Figure 9:**
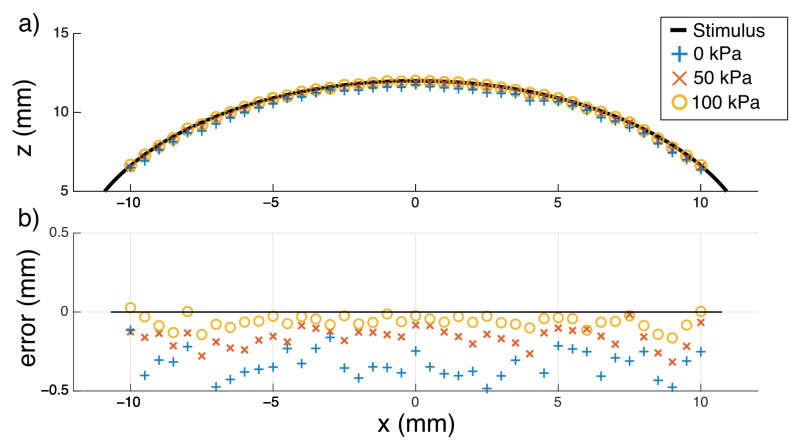
A circle fitted to the sensor’s output ideally matches the known diameter of the real soft object. (**a**) Reconstruction of a section through centre (y = 0) of a sphere of known radius (12 mm). The back-pressure states at 0, 50, and 100 kPa returned calculated radii of 11.66 mm, 11.84 mm, and 11.94 mm, respectively. (**b**) Plotting just the error shows that the higher pressure state is more accurate at sensing the edge of the soft object, but all states consistently return an error of less than half a millimetre.

The precision of shape reconstruction is dependent on the stiffness of the stimuli being detected; if the stimulus is significantly softer and more compliant than the ESPRESS.0’s silicone membrane, then it may deform more than the sensor tip. We tested stimuli with two different values of hardness. The stimuli consisted of objects made from PLA (80 D hardness [48], hard stimuli) and cast from silicone (DragonSkin 10, Smooth-on, 10 A hardness [49], soft stimuli) (Figure 8). Hard stimuli were 3D printed directly onto the testing surface, where they could be palpated, whereas the soft stimulus was cast from the 10 A silicone (DragonSkin 10, Smooth-on) using a 3D printed mould. The soft stimulus was shaped as a spherical cap, so that a circle could be fitted to the shape in the plane perpendicular to the xy plane along any chord of the shape’s 2D circular projection in that plane. This physical radius should be reflected exactly in the tactile data. The radii of the circles extrapolated from the data using the Landau-Smith method [50] were compared to the radius of the mould used to make the silicone stimulus in order to assess the accuracy of the sensory data compared to the real object (Figure 9).

**Figure 10:**
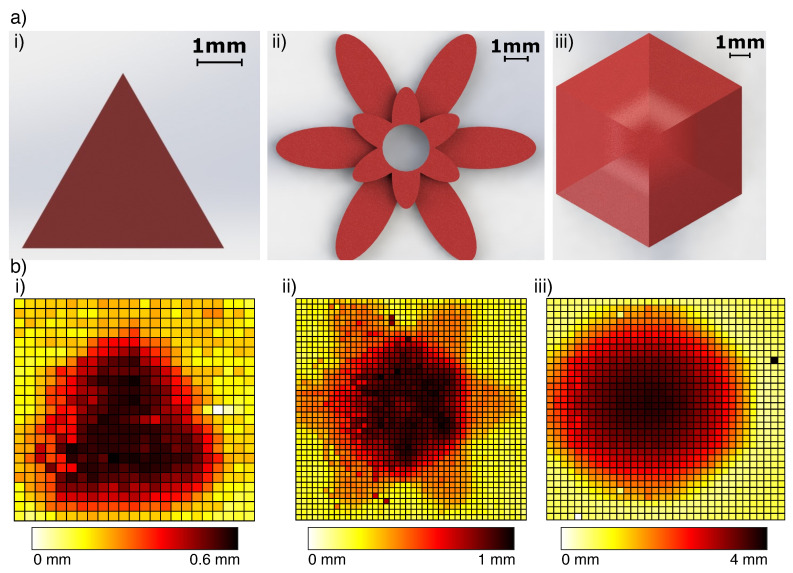
3D printed PLA models were palpated and reconstructed using a simple signal threshold. (**a**) CAD models 3D printed for the stimulus: (i) 0.6mm high triangle, (ii) flower model with 2-step height of 0.5 and 1 mm, (iii) a hexagonal dome with steep edges reaching 4mm height. (**b**) Tactile reconstruction of the shapes after a single pass, where each cell on the grid represents a 0.5 mm × 0.5 mm palpated region and the colourbar shows the elevation from the test surface: (i) triangle, (ii) flower, (iii) dome.

**Hard Stimuli:** The tip was able to detect and reconstruct features of the shapes on the sub-millimetre scale (Figure 10). Changing the back-pressure of the sensor made little difference to the ability to sense a hard object’s shape. At each pressure, the sensor was capable of finding the z-distance of each object to within the precision of the Cartesian robot (0.03 mm).

**Soft Stimuli:** Circles fitted to the tactile data across all three tested back-pressures were consistent with the 12 mm radius of the silicone stimulus and with a spherical geometry in general (Figure 9). When pre-stressed with a back-pressure of 100 kPa, the ESPRESS.0 returned the smallest error, as predicted by the steep, sensitive region in the modelling for a 2.5 mm membrane in Figure 6.

### 4.2. Stiffness Classification

**Figure 11:**
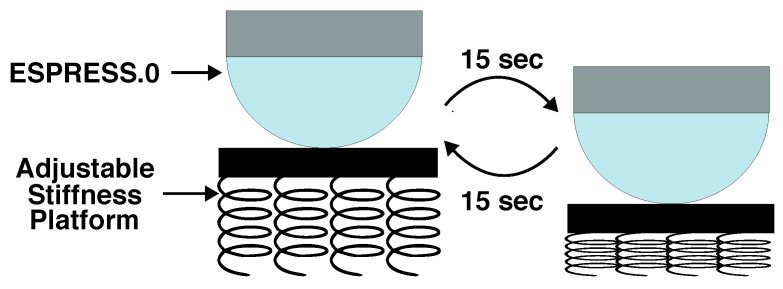
Compression of the palpation platform. The number of springs may be altered to change the linear stiffness of the platform.

In order to test an object of known and linear stiffness, a platform was constructed to be supported by a matrix of 12 springs. The springs were of identical length (53 mm) and spring constant (0.0905 N/mm), such that they could be added in parallel to adjust the spring constant of the platform as a whole. A 30 s palpation, axial to the spring base, consisting of both compression and relaxation of the base, was carried out for bases consisting of 1, 2, 3, 4, 8, and 12 springs (Figure 11). To characterise the sensor’s response to stimuli of varying stiffnesses, ten compressions were carried out. The position and force readout of the sensor were recorded at 0 and 100 kPa nominal back-pressures for a 2.5 mm membrane ESPRESS.0 tip over the range of six platform stiffnesses as well as an effectively rigid, hard object.

After characterisation, data from four continuous palpations at a speed of 25 mm/s were used to assess the capability of the ESPRESS.0 to classify the stimulus stiffness according to the sensory output. The classification algorithm fits a linear function to the characterisation data at the appropriate palpation depth and back-pressure state, and uses a least squares method to determine which characteristic line the test data lies closest to.

It is advantageous to be able to classify the stiffness properties of a stimulus using as little movement and data as possible, so that the tactile exploration is not at risk of damaging a sample and minimises discomfort and time taken for an in vivo examination such as in the case of medical diagnosis. This is particularly important when considering the task of finding deeper, smaller, or heterogeneous stiff nodes, where only a small amount of data may be possible.

The data were analysed using samples of varying sizes up to a maximum palpation depth of 5 mm. The smallest sample size corresponds to splitting the data collected into 0.3 mm slices, and the largest size corresponds to 2.1 mm.

The data show that the classifier (Table 1) is consistently more accurate if given larger samples, although for shallow palpations (≤5 mm) 0.3 mm of data is still enough to reliably classify the spring constant of the stimulus to within ≈ 0.12 N/mm (2 springs), or elastic modulus ≈ 0.8 MPa, approximately the range of human skin stiffness [51]. This would be especially useful for classifying small nodes, which may only be detectable in a small volume.

Increasing the nominal pressure state of the sensor to 100 kPa increased the ability to correctly identify the softest stimuli more reliably (from 36%, 26%, 86% to 86%, 98%, 93%, respectively), where the inverse was true for the stiffest stimuli (from 85%, 92%, 100% to 77%, 89%, 80%, respectively). At the 100 kPa state, the silicone membrane of the ESPRESS.0 sensor is near its most easily deformed for small force contacts (Figure 5 and Figure 6), and so it more readily deforms compared to a stiff stimuli. The effective stiffness (force to meniscus signal ratio) of the 100 kPa ESPRESS.0 is approximately 0.56 N/mm for forces under 3 N, in the order of the softest stimuli used here (1–4 springs is a range of 0.0905–0.3620 N/mm), where the 0 kPa ESPRESS.0 has an effective stiffness of approximately 2.02 N/mm (8–12 springs is a range of 0.7240–1.0860 N/mm). This suggests that there is a threshold upper bound on the stiffness that the ESPRESS.0 tip is useful for classifying that is near its own stiffness. Beyond this, the tip itself compresses significantly such that the stimuli are less easily distinguished. Periodically matching the ESPRESS.0’s internal pressure state, in a manner similar to the Eustachian Tube [24,52], therefore, is a viable method to maximise tactile information gain from a palpation.

### 4.3. 3D Tactile Map of Synthetic Tissue

Once the boundary of an object has been detected, a palpation can be carried out to determine the stiffness of the object in that location. The utility of this was demonstrated using a 45 mm deep tissue-mimicking sample (Polycraft Silskin 10 silicone rubber) with an elastic modulus of 12 kPa, embedded with a hard synthetic spherical node of 25 mm diameter at its base. Thus, the node had a minimum depth of 20 mm from the surface at its closest point, and a maximum of 32.5 mm at its equator. In this controlled test case, the range of expected stiffnesses was known and so the untethered ESPRESS.0 (Section 2.1.2) was used to demonstrate the potential for this platform to be used to carry out untethered palpations. The untethered ESPRESS.0 carried out a palpation in the z-axis at the centre of each mm${}^{2}$ coordinate in the xy plane over a predefined area of the sample. This particular palpation technique was selected for its simplicity and repeatability in order to demonstrate the suitability of the ESPRESS.0 to the task of embedded lump detection and localisation.

The untethered ESPRESS.0 made a vertical movement downward until it detected a stimulus, then moved by a further 15 mm, followed by a return to the original position upwards. The 15 mm depth relates to the depth of the untethered ESPRESS.0 tip’s position, where it may detect a change in stiffness due to an object that is much deeper within the sample. The untethered ESPRESS.0 repeated this movement at each point on a 20 mm × 20 mm grid in steps of 1 mm in order to palpate the whole region. The palpation manoeuvre was set to take just under 2 s, such that the examination of the 20 mm × 20 mm area down to a 15 mm depth took 12 min. This speed allows the Raspberry Pi Zero to reliably stream its video capture to the PC wirelessly in high quality. Stiffness values were generated following the technique set out in Section 4.2, and the data were used to generate an image for each mm depth-slice of the data using the stiffness values at each coordinate, illustrating the stiffness profile and tactile information of the region of the sample. Each slice of the data was thresholded at the mean for the area, such that the remaining area above this threshold could be used to infer the edge of any detected embedded object, and the centroid of the object calculated using a centre of mass approach for the signal over the remaining area.

**Figure 12:**
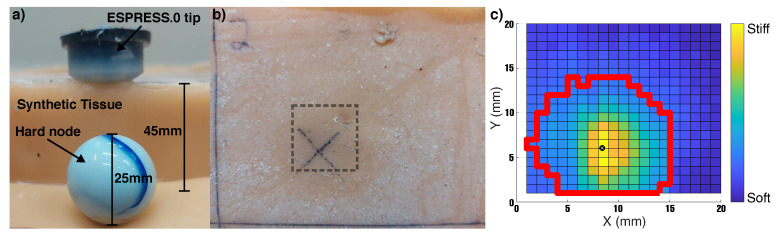
Palpation of synthetic tissue sample with embedded hard node. (**a**) Illustration of depth and size of components involved and synthetic tissue. (**b**) Top view of the sampled area on the synthetic silicone tissue sample. (**c**) The top view of tactile output at 15mm depth shows the centroid of the stimulus (white cross in black circle) and its inferred shape (red line).

Figure 12 shows the deepest slice output by the tactile exploration of the tissue mimicking sample. The Figure represents the stiffness encountered at 15 mm depth at each xy coordinate. The maximum stiffness encountered corresponds to a contact force of 4.9 N, or a contact pressure of 16 kPa, which is far below the suggested threshold for patient discomfort during mammography examinations (50–90 kPa [53]) and is in the order of target pressures for completely comfortable examinations (8–13.9 kPa [54]).

The analysis returned that the untethered ESPRESS.0 had detected a stimulus of roughly circular profile with a diameter of 15 mm and a symmetric stiffness profile that peaks in the centre.

## 5. Discussion

### 5.1. Shape Reconstruction and Super Resolution

Using a super-resolution method of overlapping taxels from multiple passes, ESPRESS.0 can detect shapes and their surface features on a sub-millimetre scale. Using multiple passes to generate a super resolution for a taxel-based sensor has been explored thoroughly for surface features in the literature, where a visual comparison may be used to validate the improvement gained from the super-resolution method [55,56]. In this work, we have demonstrated that the super-resolution method can be extended into detecting tactile heterogeneity within a 3D sample, where the ≈12.5 mm${}^{2}$ exposed surface area of a single sensitive channel of the ESPRESS.0 produced a 2D cross-section of the tactile map for a synthetic tissue sample with a resolution of 1 mm${}^{2}$. This meant that a simple analysis of the sensor output could be used to locate the centroid of a hard inclusion buried deep within a soft synthetic tissue matrix.

### 5.2. Stiffness Classification

Integrating location information with the sensor transforms the system from a force sensor to a palpation platform, and tracing a stimulus’ mechanical deformation as a function of applied force means that its stiffness can be inferred.

Utilising the ESPRESS.0’s actuation mechanism and pre-stressing the membrane by applying an internal pressure allows it to be tuned to a range of applied forces. This becomes useful for a stiffness classification task when the sensor is of a similar stiffness to the stimulus. If the sensor is an order of magnitude softer than the stimulus, then the stimulus will not deform appreciably, effectively making it indistinguishable from a completely rigid object, and if the sensor is an order of magnitude stiffer, then signals from interactions with softer stimuli are too subtle to distinguish between them.

The classification can make a reasonable estimate of the stiffness of a stimulus following a palpation of only 0.3 mm depth. This is particularly of importance where the palpation of a stimulus is limited (e.g., if it is causing pain, the stimulus is very small, or if the stimulus has high heterogeneity) and, hence, a classifier would have a smaller amount of data to use. The latter is a particular focus of recent research in the area of breast cancer [39,40,41,42]. Optimising a sensor and analysis to work with as small a sample of palpation data as possible is uncommon in the literature. Still, it enhances the generalisability and the efficiency of the system and, hence, illustrates enhanced sensitivity.

An ESPRESS.0 whose signal has been saturated, i.e., the applied force is higher than its working range, can recover sensitivity if a back-pressure is applied ad hoc. It was demonstrated to recover a level of sensitivity to normal forces as small as 0.006 N in the range of large applied forces around 40 N. This sensitivity was not explicitly maximised; it is expected that ESPRESS.0 could become more sensitive than this, but the silicone membrane could become susceptible to tearing or rupturing as it approaches its mechanical material limits.

### 5.3. 3D Tactile Map of Synthetic Tissue

The small size of the ESPRESS.0 and its low power demand make it suitable for use as an untethered sensor, here demonstrated with its use for a synthetic tissue palpation task. Untethered tactile sensors may more easily be integrated with existing systems compared to tethered alternatives. Multiple small sensors may act simultaneously over a larger area to maximise the efficiency of a tactile examination, which would be particularly useful for medical palpation diagnosis.

To the authors’ knowledge, this work is the first targeting depth of inclusions at 20 mm, with comparable studies in the literature using nodes at shallower depths (Table 2).

### 5.4. Future Work

Future work includes developing an adaptive active controller to maintain an appropriate level of sensitivity such that as the ESPRESS.0 tip is impressed into a stimulus, the sensory response is adjusted to lie within a predetermined range. This range would determine a lower and upper threshold as appropriate to the task. If the response is under the lower threshold or over the upper threshold, the ESPRESS.0’s nominal pressure would be altered to soften or stiffen the tip, respectively. This would ensure that the ESPRESS.0 maintains an appropriate level of sensitivity to its stimulus, much like the mammalian Eustachian tube controls the eardrum’s sensitivity to stimuli [24,25,26].

In order to make the full ESPRESS.0 system untethered with the ability to adjust its internal pressure and thus gain the benefits of sensitivity and range adjustment, the pressure controlling actuator would have to be miniaturised. This might include using a small pump, syringe driver, or peristaltic pump. Here, we have separately demonstrated the benefits of internal pressure adjustment and the feasibility of an untethered sensor. Combining these two properties is goal for future research.

The capability of ESPRESS.0 was demonstrated to distinguish between stimuli of different stiffnesses, and separately for a single embedded node of fixed stiffness. For the sensor to be viable as part of a system for medical palpation, future work will seek to be demonstrate that it can usefully distinguish between embedded nodes of different stiffnesses. Platforms that are able to present a phantom with embedded nodes of variable stiffnesses such as [57] show promise to enable future research that aims to achieve this.

There is a maximum velocity with which liquid can move through the tubes before a turbulent flow means that air bubbles start to be introduced, causing a significant discontinuity in the sensor readout. To avoid this, the system was operated at velocities far below this threshold. Future work would include theoretical and experimental evaluation of this velocity so that versions of the sensor may be designed with this operational speed as a design parameter.

There remains a significant gap between the tasks demonstrated here and those needed for a fully autonomous palpation: speed and comfort would need to be assessed more explicitly. Live subjects provide a more difficult tactile stimulus due to dynamic conditions such as actively moving, breathing, and presenting a much larger set of mechanical stimuli when stationary. Enabling the other sensory channels and characterising the sensory response in different environments, e.g., under different temperatures, and demonstrating that the sensor can be scaled to different sizes and geometries are important next steps for the characterisation of the sensor.

## 6. Conclusions

This paper has presented a novel tactile device that is capable of automatic palpation in order to sense the shape and stiffness of a stimulus. Its particular potential lies in modifying its compliance in order to maximise sensitivity for different stimuli, including sensing forces that might appear to be outside its normal working range. The modelling of the sensitive membrane when internally pressurised and the complementary experiments demonstrate that the hyper-elastic stretching of the membrane dominates the mechanical response of the sensor.

The potential of the ESPRESS.0 system to carry out palpation examinations, as well as to store and display the results make it a good candidate for use as part of an automated lump detection system. The working principle behind the sensor and its ability to adjust its stiffness ad hoc for different stimuli makes it well suited for application as a general purpose tactile sensor, where a single device may be suitable for a range of applications.

.

## Figures and Tables

**Table 1:** Confusion Matrices for low and high pressure states of a 2.5 mm membrane ESPRESS.0 classifying the stiffness of a springboard based on the signal from small or large data samples. Green indicates correct classifications, while significantly populated (≥0.1) misclassifications are highlighted in a shade of red, the intensity of which is relative to the cell’s distance from the diagonal.

Palpation with small (0.3 mm) sample at 0 kPa										Palpation with large (2.1 mm) sample at 0 kPa								
		Number of springs (0.0905 N/mm) classified										Number of springs (0.0905 N/mm) classified						
		hard	12	8	4	3	2	1				hard	12	8	4	3	2	1
Actual	hard	1.00	0.00	0.00	0.00	0.00	0.00	0.00		Actual	hard	1.00	0.00	0.00	0.00	0.00	0.00	0.00
	12	0.00	0.92	0.08	0.00	0.00	0.00	0.00			12	0.00	0.85	0.15	0.00	0.00	0.00	0.00
	8	0.00	0.13	0.33	0.30	0.12	0.06	0.06			8	0.00	0.01	0.92	0.08	0.00	0.00	0.00
	4	0.00	0.07	0.17	0.26	0.21	0.09	0.20			4	0.00	0.00	0.00	1.00	0.01	−0.01	0.00
	3	0.00	0.01	0.08	0.34	0.29	0.10	0.18			3	0.00	0.00	0.00	0.25	0.36	0.39	0.00
	2	0.00	0.00	0.03	0.07	0.24	0.09	0.56			2	0.00	0.00	0.00	0.06	0.40	0.26	0.28
	1	0.00	0.00	0.00	0.00	0.21	0.13	0.67			1	0.00	0.00	0.00	0.00	0.00	0.14	0.86
**Palpation with small (0.3 mm) sample at 100 kPa**										**Palpation with large (2.1 mm) sample at 100 kPa**								
		Number of springs (0.0905 N/mm) classified										Number of springs (0.0905 N/mm) classified						
		hard	12	8	4	3	2	1				hard	12	8	4	3	2	1
Actual	hard	0.91	0.09	0.00	0.00	0.00	0.00	0.00		Actual	hard	1.00	0.00	0.00	0.00	0.00	0.00	0.00
	12	0.02	0.88	0.05	0.07	0.00	0.00	−0.01			12	0.00	0.77	0.23	0.00	0.00	0.00	0.00
	8	0.03	0.04	0.63	0.29	0.01	0.01	0.00			8	0.00	0.08	0.89	0.03	0.00	0.00	0.00
	4	0.00	0.02	0.03	0.49	0.30	0.15	0.01			4	0.00	0.00	0.00	0.80	0.20	0.00	0.00
	3	0.00	0.02	0.00	0.20	0.69	0.06	0.02			3	0.00	0.00	0.00	0.06	0.86	0.08	0.00
	2	0.00	0.00	0.00	0.06	0.44	0.54	0.18			2	0.00	0.00	0.00	0.00	0.02	0.98	0.00
	1	0.00	0.01	0.00	0.03	0.06	0.25	0.65			1	0.00	0.00	0.00	0.00	0.00	0.07	0.93

**Table 2:** This work represents the deepest hard node inclusion to be palpated in the reviewed literature.

Study	Hard Node Depth
Gwilliam et al. (2010) [3]	3.5 mm
Yamamoto et al. (2009) [7]	5 mm
Konstantinova et al. (2017) [31]	5 mm
Konstantinova et al. (2014) [32]	5 mm
Garg et al. (2016) [5]	8 mm
Sonrkarn et al. (2017) [33]	8 mm
Herzig et al. (2020) [19]	8 mm
Ly et al. 2021 [34]	8 mm
Konstantinova et al. (2014) [35]	11 mm
Scimeca et al. (2020) [36]	15 mm
Sangpradit et al. (2011) [37]	15 mm
**This work**	20 mm

## Data Availability

The data presented in this study are openly available at https://github.com/geojenks/espresso (accessed on 18 November 2022).
